# Blood pressure tracking in urban black South African children: birth to twenty cohort

**DOI:** 10.1186/s12887-015-0402-z

**Published:** 2015-07-15

**Authors:** Juliana Kagura, Linda S Adair, Mogi G Musa, John M Pettifor, Shane A Norris

**Affiliations:** Department of Paediatrics and Child Health, MRC/Wits Developmental Pathways for Health Research Unit, University of the Witwatersrand, 7 York Road, Johannesburg, 2193 South Africa; Department of Nutrition, University of North Carolina, Chapel Hill, NC USA

**Keywords:** Paediatric, Blood pressure, Hypertension, Tracking

## Abstract

**Background:**

Hypertension is an emerging public health problem in South Africa. Recent evidence from longitudinal studies has shown that hypertension in adulthood can be traced back to childhood. There is scarcity of longitudinal data on paediatric blood pressure (BP) particularly in African populations. The objective of this study is to assess the prevalence of hypertension and evaluate BP tracking between childhood and late adolescence among South African black Children.

**Methods:**

This study utilized data from the Birth to Twenty cohort, which is comprised of children born in Soweto, Johannesburg in 1990 (N = 3273, 78.5 % black). Data on BP and anthropometry were collected at six follow-up periods between ages 5 and 18 years. Blood pressure status was classified using the Fourth report on National High Blood pressure program in children and adolescents. Pearson correlation coefficients and relative risk ratios (RR) were used to describe tracking of BP between childhood and late adolescence.

**Results:**

The overall point prevalence ranged from 9.2 to 16.4 % for prehypertension and 8.4 to 24.4 % for hypertension. Tracking coefficients ranged from 0.20 to 0.57 for SBP and 0.17- 0.51 for DBP in both sexes over the 14 years of measurement. The proportion of children who maintained an elevated BP status between childhood, adolescence and age 18 years ranged from 36.1 % at age 5 years to 56.3 % at age 13 years. Risk of having elevated BP at 18 years ranged from; RR: 1.60 (95 % CI: 1.29–2.00) at 5 years to RR: 2.71 (95 % CI: 2.32–3.17) at 14 years of age.

**Conclusions:**

This study reports high prevalence of elevated BP which tracks from early childhood into late adolescence. These findings emphasize the importance of early identification of children at risk of developing elevated BP and related risk factors plus timely intervention to prevent hypertension in adulthood.

## Background

The increasing prevalence of childhood and adolescent hypertension is rapidly emerging as a global public health concern [1, 2]. Although the condition was rarely diagnosed in children due to the lack of its inclusion during routine screening, there is now growing evidence to suggest that essential hypertension in adulthood has its roots in childhood and adolescence [3, 4]. Epidemiological evidence from longitudinal cohorts such as the Young Finns study [5], Bogalusa Heart study [6], Muscatine study [7] and the Kangwha study [8] showed that BP tracks from childhood to adulthood. In line with this, a systematic review and meta-regression analysis of a various paediatric cohorts by Chen and Wang [9] reported moderate BP tracking from childhood to adulthood, with overall tracking coefficients stronger for systolic blood pressure compared (SBP = 0.38) to diastolic blood pressure (DBP = 0.28) with marginal sex discrepancies.

The majority of longitudinal studies following blood pressure patterns from childhood to adulthood comes from high income countries (HICs), there being a paucity of similar studies from low-middle income countries (LMICs) like South Africa where epidemiology of hypertension appears to be unique. The most recent global report from the WHO-SAGE study ranked South Africa highest in prevalence of hypertension in people aged 50 or older (78 %) [10]. The Heart of Soweto study reported a hypertension prevalence of 55 % among adults aged 52.8 years [11]. A few studies in South Africa have assessed blood pressure profiles in children and adolescents and have reported a prevalence of hypertension in black children ranging from 1 to 25.9 % [12–15]. However, these studies were cross sectional, mostly conducted in rural settings and were not designed to establish tracking of elevated blood pressure into late adolescence.

The longitudinal tracking of BP may provide insight into the aetiology of essential hypertension in adult Black South Africans. Until now, no studies have adequately addressed this because of the lack of longitudinal data on BP in children and adolescents in the same setting. The present study aims to (1) describe the prevalence of elevated BP in a population of urban black South African children and adolescents aged between 5 to 18 years, (2) assess tracking of BP from childhood to at 18 years of age.

## Methods

### Study sample

Data for this study are from the Birth-Twenty cohort, which is following singleton infants, born in the Soweto-Johannesburg Metropolis (N = 3273) in 1990. Mothers of these children were residents of Soweto recruited from antenatal clinics who were expected to deliver within the seven-week period between 23 April and 8 June 1990. Follow up was done telephonically or through field visits and contact with parents/caregivers and participants were maintained between data collection time points by newsletters and birthday cards. The details of recruitment and cohort attrition have been described elsewhere [16]. For this study, only black participants were selected who had anthropometric measurements and BP assessments in 1995 (n = 1026), 1998 (n = 1024), 2002 (n = 1351), 2003 (n = 1391), 2005 (n = 1624), 2008 (n = 1587).

### Measures

#### Anthropometry

Trained research assistants carried out anthropometric measurements. Weight was measured on a digital scale to the nearest 0.1 kg with participants in light clothes without shoes. Standing height was measured by means of a wall-mounted stadiometer (Holtain, UK) to the nearest 0.1 cm. Body Mass Index (BMI) was computed using the formula: weight (kg) divided by height squared (m^2^) and converted to WHO BMI z scores for age in STATA.

#### Blood pressure assessment

Blood pressure was measured using an Omron 6 automated machine (Kyoto, Japan) from the age 8 years onwards; while a Dinamap Vital Signs monitor 1846SX (Critikon, USA) was used at age 5 years. Three individual measurements were taken at intervals of 2 min with participants in a seated position and using an appropriate cuff size after 5 min of siting in a resting position. The first BP reading was discarded and the second and third BP measurements were averaged for all the analyses. We used age, sex and height standardized percentile tables for BP classification in children and adolescence in the analysis where BP status was classified for each gender and CDC-standardised height percentile as either normotensive (<90^th^ percentile), prehypertensive (≥90^th^ to < 95^th^ percentile or >120/80 mmHg if <90^th^ percentile) or hypertensive (≥95^th^ percentile) using the fourth report from the National High Blood Pressure Education Program working group on high blood pressure in children and adolescents (NHBPEP) [17] as a guideline.

### Statistical analyses

We used descriptive statistics to assess sex differences in variables, for comparison of participants followed up between baseline and age 18 years and to compute proportions of BP status at each time point using student *t*-test for continuous variables and chi square test for categorical variables. Pearson correlations were used to assess the tracking of BP from baseline at 5 years to the end of follow-up at 18 years. We used multinomial logistic regression models to compute relative risk ratios (RR) to assess the risk of having elevated BP at 18 years of age given an elevated BP status in childhood or adolescence with normotensive status as a reference group (RR = 1). The probability value for statistical significance was p < 0.05 unless indicated and all the analyses were done using STATA 11.

### Ethical considerations

Prior to the study, ethical approval was obtained from the Human Research Ethics Committee of the University of the Witwatersrand, Johannesburg (M130556). Informed consent was sought from parents/caregivers when the participants were minor at the beginning of each data collection session. Data on this study is available on request from the Developmental Pathways for Health Research Unit data management department.

## Results

### Study characteristics

Age and sex-stratified mean systolic (SBP), diastolic blood pressures (DBP) and BMI are presented in Table 1. Boys had a marginally higher SBP compared to girls at mean ages 16 and 18 years of age (p < 0.001). Girls tended to have slightly higher DBP compared to boys but the differences were only significant at mean ages 13 and 14 years (p < 0.001). Sex differences emerged from age 13 years to 18 years with girls having markedly higher BMI compared to boys (p < 0.001). No differences were noted between those who did and did not have information on anthropometry and BP measurements at ages 5 and 18 years, except that those who had measurements at both 5 and 18 years of age had marginally lower BMI (Appendix 1) hence BMI was adjusted in all the models.

**Table 1:** Sex differences in blood pressure and BMI in urban black South African children by mean age

Sex	Mean age (years)	Number	SBP (mmHg)	DBP (mmHg)	BMI (Kg/m^2^)
Males	5	503	108 (13)	63 (8)	16 (1)
	8	506	109 (10)	69 (8)	16 (1)
	13	643	106 (10)	65 (8)	18 (3)
	14	659	108 (11)	68 (9)	19 (3)
	16	779	117 (12)	68 (10)	20 (3)
	18	766	121 (11)	71 (9)	20 (3)
Females	5	523	108 (12)	64 (9)	16 (1)
	8	523	109 (11)	70 (9)	16 (2)
	13	708	106 (10)	67 (8)	19 (4)
	14	732	107 (10)	70 (9)	21 (4)
	16	845	110 (12)	68 (9)	22 (4)
	18	821	115 (10)	72 (9)	23 (5)

A student’s *t*-test was used for all analyses and results presented as mean (standard deviations) and level of significance set at:*P < 0.05; ***p < 0.001

### Prevalence of prehypertension and hypertension

Table 2 shows the prevalence of pre-hypertension and hypertension at the various time points in childhood and adolescence. The prevalence of prehypertension remained fairly constant across the ages, ranging from 9.2 % at age 8 years to 16.4 % at age 16 years in the sexes combined. The prevalence of hypertension in boys and girls combined ranged from 22.4 % at age 5 years to 15.7 % at age 18 years, with the prevalence falling in the intervening years. Sex differences in BP status only emerged at age 16 years, with the proportions of boys having elevated BP being higher than girls (p < 001).

**Table 2:** Sex-specific blood pressure status in urban black South African children aged 5 to 18 years

Age (years)	Sex	BP status		
		Normotensive N (%)	Pre hypertensive N (%)	Hypertensive N (%)
5	M	353 (70.2)	52 (10.3)	98 (19.5)
	F	342 (65.4)	49 (9.4)	132 (25.2)
	Total	695 (67.8)	101 (9.8)	230 (22.4)
8	M	334 (66.3)	44 (8.7)	126 (25.0)
	F	348 (66.4)	51 (9.7)	125 (23.9)
	Total	682 (66.3)	95 (9.2)	251 (24.4)
13	M	490 (82.9)	63 (10.7)	38 (6.4)
	F	522 (79.6)	67 (10.2)	67 (10.2)
	Total	1015 (81.3)	130 (10.4)	105 (8.4)
14	M	502 (76.1)	75 (11.4)	83 (12.6)
	F	548 (74.9)	92 (12.6)	92 (12.6)
	Total	1050 (75.4)	167 (12.0)	175 (12.6)
16	M	466 (59.7)	172 (22.1)	142 (18.2)***
	F	653 (77.2)	94 (11.1)	99 (11.7)
	Total	1119 (68.8)	266 (16.4)	241 (14.8)
18	M	552 (72.0)	96 (12.5)	119 (15.5)
	F	595 (72.2)	98 (11.9)	131 (15.9)
	Total	1147 (72.1)	194 (12.2)	250 (15.7)

A chi square test was conducted to describe the gender differences in blood pressure status at each age category. Some proportions do not add up to 100 % because of rounding off to the nearest 0.1 percentage

### Tracking of blood pressure

Tracking coefficients are presented in Table 3 with the average BMI-adjusted correlation coefficients between ages 5 and 18 years for SBP and DBP ranging from 0.20 to 0.57 and 0.17 to 0.51, respectively. It is noteworthy that all the tracking coefficients remained significant even after adjusting for BMI. Overall, the mean tracking coefficients for SBP were marginally higher than those for DBP with inconsistent sex differences in the multivariate models.

**Table 3:** Sex specific tracking correlation coefficients for blood pressure from 5 to 18 years of age

Correlation between two mean age categories	Systolic BP				Diastolic BP			
	Boys		Girls		Boys		Girls	
	Crude	Adjusted	Crude	Adjusted	Crude	Adjusted	Crude	Adjusted
5–8	0.26***	0.22***	0.39***	0.33***	0.19***	0.18***	0.21***	0.17***
5–13	0.28***	0.20***	0.27***	0.20***	0.24***	0.21***	0.20***	0.17***
5–14	0.25***	0.20***	0.16***	0.16***	0.22***	0.22***	0.15***	0.13***
5–16	0.22***	0.20***	0.25***	0.23***	0.23***	0.26***	0.26***	0.25***
5–18	0.23***	0.19***	0.18***	0.13**	0.23***	0.25***	0.15***	0.14**
Average	0.25	0.20	0.30	0.21	0.22	0.22	0.19	0.17
8–13	0.42***	0.38***	0.43***	0.38***	0.25***	0.20***	0.32***	0.26***
8–14	0.42***	0.41***	0.43***	0.39***	0.28***	0.31***	0.36***	0.36***
8–16	0.27***	0.30***	0.40***	0.44***	0.18***	0.17***	0.31***	0.30***
8–18	0.29***	0.29***	0.34***	0.29***	0.22***	0.22***	0.27***	0.28***
Average	0.35	0.35	0.40	0.38	0.23	0.23	0.32	0.30
13–14	0.53***	0.61***	0.60***	0.61***	0.53***	0.55***	0.58***	0.62***
13–16	0.50***	0.57***	0.42***	0.49***	0.39***	0.43***	0.41***	0.44***
13–18	0.50***	0.53***	0.41***	0.40***	0.36***	0.39***	0.47***	0.49***
Average	0.51	0.57	0.47	0.50	0.42	0.46	0.49	0.51
14–16	0.53***	0.57***	0.47***	0.54***	0.48***	0.51***	0.46***	0.49***
14–18	0.47***	0.45***	0.54***	0.44***	0.39***	0.39***	0.48***	0.48***
Average	0.50	0.51	0.51	0.49	0.43	0.45	0.47	0.49
16–18	0.46***	0.36***	0.44***	0.34***	0.37***	0.34***	0.45***	0.42***

Simple and multiple linear regressions adjusted for BMI-age z score. Significant at **p < 0.01 ***p < 0.001

### Relative risk of having elevated BP status at age 18 years

Table 4 provides the relative risk ratios at each age category for having elevated BP in late adolescence. BP status was classified as normotensive (<90^th^ percentile) or elevated BP (combining prehypertension and hypertension groups). There is a consistent, significant relationship between BP status at each of the ages 5, 8, 13, 14, 16 years and that in late adolescence (p < 0.001). Between a third and a half of children who had elevated BP at ages 5, 8, 13, 14 and 16 years, remained with elevated BP status at 18 years, while approximately 20 % of children who were normotensive at each age group had elevated BP at age 18 years. The majority of the participants remained normotensive between an earlier time point and age 18 years (range: 77–80 %) while 43.8 % (age 5 years) to 63.9 % (age 13 years) of those who had elevated BP became normotensive at age 18 years.

**Table 4:** Relative risk of elevated BP in urban black South African children between childhood and late adolescence

Mean Age (years)	BP Status	BP status at 18 years			Relative Risk (95 % CI)	
		Normotensive	Elevated BP	Total	Crude	BMI-adjusted
5	Normotensive	461 (77.3)	135 (22.7)	596 (69.4)***	1 (ref)	1 (ref)
	Elevated BP	168 (63.9)	95 (36.1)	263 (30.6)	1.60 (1.28–2.00)	1.60 (1.29–2.00)
	Total	629 (73.2)	230 (26.8)	859 (100.0)		
8	Normotensive	454 (77.2)	134 (22.8)	588 (66.7)***	1 (ref)	1 (ref)
	Elevated BP	184 (62.8)	109 (37.2)	293 (33.3)	1.75 (1.43–2.14)	1.69 (1.39–2.06)
	Total	638 (72.4)	243 (27.6)	881 (100.0)		
13	Normotensive	719 (78.4)	198 (21.6)	917 (81.5)***	I (ref)	1 (ref)
	Elevated BP	91 (43.8)	117 (56.3)	208 (18.5)	2.70 (2.28–3.20)	2.56 (2.16–3.04)
	Total	810 (72.0)	315 (28.0)	1125 (100.0)		
14	Normotensive	770 (80.1)	191 (19.9)	961 (75.2)***	1 (ref)	1 (ref)
	Elevated BP	147 (46.5)	169 (53.5)	316 (24.8)	2.84 (2.43–3.32)	2.71 (2.32–3.17)
	Total	917 (71.8)	360 (28.2)	1277 (100.0)		
16	Normotensive	830 (80.0)	207 (20.0)	1037 (69.4)***	1 (ref)	1 (ref)
	Elevated BP	246 (53.7)	212 (46.3)	458 (30.6)	2.34 (2.01–2.72)	2.28 (2.00–2.65)
	Total	1076 (72.0)	419 (28.0)	1495 (100.0)		

Chi square test was used to assess the difference in BP status at a given two time points and results presented as proportions: n (%). Some proportions do not add up to 100 % because of rounding off to the nearest percentage and level of significance set at ***P < 0.001

The relative risk of having an elevated BP at age 18 years for children or adolescents with BP above the 90^th^ percentile for their age, sex and height is presented in Table 4. Children who had an elevated BP at each of the age categories had risk of between 1.60 (95 % CI: 1.29 to 2.00) and 2.71 (95 % CI: 2.32 to 3.17) of having an elevated BP at 18 years of age compared to their normotensive peers (p < 0.001 in all adjusted models). Adjusting for BMI only reduced the risk slightly.

## Discussion

The present study reports a high prevalence of elevated BP, which persists into late adolescence, in a longitudinal cohort of urban black South African children and adolescents. Approximately one-third to a half of children who were hypertensive at some time during childhood and adolescence where hypertensive at age 18 years. The risk of having elevated BP at 18 years of age was lowest at age 5 years and highest at age 14 years.

We reported an overall prevalence of hypertension (>95^th^ percentile) of 22.4 % and 24.4 % at ages 5 and 8 years, respectively. The prevalence of hypertension in children and adolescents reported in other studies varied considerably from 0 % to as high as 25.9 % in children aged 6 to 15 years of age [15, 18, 19]. A study from the North-West province of South Africa reported a prevalence of hypertension of 25.9 % in Grade one learners aged 6 years based on one visit and an average of three BP measurements classified according to NHBPEP [14]. Studies from other countries in the Sub Saharan Africa have reported a lower prevalence of hypertension in children than the current study; Zambia (6.9 % in boys and 6.1 % in girls) [20], Congo (10.1 %) [21], Nigeria (4.7 %) [22]. This could possibly be attributed to the epidemiological transition taking place in South Africa and which is characterized by a shift from under-nutrition to overnutrition, and by refined foods and high salt intake in children and adolescence which are risk factors for elevated BP in children, but this needs to be tested within our population [23]. It is noteworthy that though many studies from HICs have reported a prevalence of hypertension in children as low as 3 % [24, 25], it is reported that the prevalence of hypertension is high in Hispanic (23.6 %) and African-American (17,2 %) populations aged between 8 and 13 years [19] which is comparable to our study.

Our results suggest that there is a positive weak-to-moderate tracking of BP in this cohort which is comparable to other studies; with adolescent baseline BP tracking better compared to childhood BP [26, 27]. Similar to our findings, Bao and colleagues [27] reported tracking coefficients varying from 0.36 to 0.50 for SBP and 0.20 to 0.42 for DBP in children ages 5 to 14 years after a 15 year-follow-up period, with BMI having no effect on the degree of tracking. Similarly, Toschke et al [28] reported a pooled tracking coefficient of 0.37 from 29 cohort studies of children aged 10 to 20 years. Consistent with other studies, we found out that SBP tracked better than DBP [29, 30]. Clarke et al [30] reported tracking coefficients in SBP and DBP of 0.30 and 0.18, respectively.

Existing evidence on sex differences in BP tracking is conflicting; some studies have shown no major differences [29, 31] whereas others have reported that SBP track better in boys (0.38) compared to girls (0.30) but girls (0.22) have higher tracking coefficients compared to boys (0.20) for DBP [32]. A previous review on blood pressure tracking from childhood to adulthood found little sex differences in BP tracking with men having stronger tracking for SBP and DBP [9]. We have shown that sex differences in BP tracking become more pronounced in adolescent baseline ages compared to childhood; with SBP tracking better in boys than girls and DBP tracking better in girls than in boys. The mechanism underlying these sex discrepancies in BP tracking before or during adolescence are not fully understood and require further research though some authors have suggested that pubertal development might influence BP tracking especially in adolescence [33].

We found that about 36 % of the children who had elevated BP at ages 5 or 8 years retained the elevated BP status at age 18 years of age. These findings are consistent with a recent report from a Germany study of 13 261 both sick and healthy children aged between 3 and 21 years, attending an outpatient clinic, in which the persistence of elevated blood pressure was as high as 35.6 % in a six-year follow-up study [34]. Similar results have been reported from the USA on data from the National Childhood Blood Pressure database [35]. The Bogalusa Heart Study of children initially aged 5-14 years found that of those who were clinically diagnosed with hypertension after 26 years, 48 % and 41 % had elevated SBP and DBP at baseline, respectively [26].

Some longitudinal studies used odds and hazard ratios (HR) to assess the association between an earlier BP status and the end of follow up BP status (>90^th^ percentile). For instance, the Fels Longitudinal study reported that the odds of having elevated BP as an adult aged 30 years or above given a childhood elevated BP status (5 to 7, 8 to 13 or 14 to 18 years of age) ranged from 1.8 to 4.4 [36]. A school-based screening program from Texas, USA reported that the likelihood of having hypertension after a 2-year follow up in children aged 10 to 19 years was almost 5-fold; HR:4.89 (95 % CI:1.48-16.19) [37].

Consistent with previous studies, girls had significantly higher mean DBP than boys at ages 8, 13, 14 and 18 years; but boys had higher SBP than girls between ages 14 and 18 years and these sex differences became more apparent as the adolescents grew older [38]. BMI was considered as a covariate in tracking of blood pressure. Sex differences emerged in BMI in adolescence with girls having higher BMI compared to boys.

A positive feature of this study is its longitudinal design which has confirmed in a LMIC setting results similar to those reported in HICs. We also made use of automated BP monitors, which reduce observer bias compared to the sphygmomanometer that has been reported to be prone to subjectivity in detecting the Korotkoff sounds in children [39]. Furthermore, we classified BP status by sex, height and age of the child, which are the main confounders of BP status in growing individuals. We acknowledge that our study had a limitation in that BP classification was different to the NHBPEP recommendations that children and adolescents should only be classified as pre-hypertensive or hypertensive based on an average BP from three measurements on three different occasions in the same year.

## Conclusions

This longitudinal study of BP in urban black South African children confirms the existence of elevated BP in a significant proportion of children and adolescents. We also reported that the majority of children (77 to 80 %) who were designated normotensive remained in that status showing that in as much as it is vital to identify the children at risk of having persistent elevated BP, it is also important to fully assess the profiles of the children who remain normotensive or who had elevated BP initially but reverted to the normotensive status at 18 years of age to inform early life intervention programs. The implications of these findings are that early identification of children with elevated BP and associated risk factors through pediatric screening programs and timely interventions could be vital to reduce the risk of elevated BP in adulthood. In addition, more research is required to assess the feasibility and the cost effectiveness of the screening and intervention programs in LMICs such as South Africa.

## Appendix 1

**Table 5:** Comparison of the study sample that had BP status measured at ages 5 and 18 years and those who did not

Baseline variables (5 years)	Number	BP status at both ages 5 and 18 years	Number	No BP status at both ages 5 and 18 years	P value
SBP (mmHg)	895	108 ± 13	7	109 ± 15	0.8524
DBP (mmHg)	859	63 ± 8	7	812 (64 ± 8)	0.6664
Height (cm)	859	107.8 ± 4.5	80	812 (106.9 ± 3.2)	0.0722
Weight (kg)	859	18.3 ± 2.3	210	812 (18.2 ± 2.0)	0.6855
BMI	859	15.6 ± 1.3	80	812 (16.1 ± 1.6)	0.0012
Sex: Males (%)	429	50.0	406	50.0	0.981
Females (%)	430	50.1	406	50.0	

*t*-test and chi square test conducted for continuous variables and categorical variable, respectively

## Abbreviations

SBP: Systolic blood pressure
DBP: Diastolic Blood pressure
